# Reading charts in ophthalmology

**DOI:** 10.1007/s00417-017-3659-0

**Published:** 2017-04-14

**Authors:** W. Radner

**Affiliations:** Austrian Academy of Ophthalmology, Mollgasse 11, 1180 Vienna, Austria

**Keywords:** Reading acuity, Reading charts, Reading performance, Reading speed, Sentence optotypes

## Abstract

A new generation of logarithmic reading charts has sparked interest in standardized reading performance analyses. Such reading charts have been developed according to the standards of the International Council of Ophthalmology. The print size progression in these calibrated charts is in accordance with the mathematical background of EN ISO 8596. These reading charts are: the Bailey–Lovie Word Reading Chart, the Colenbrander English Continuous Text Near Vision Cards, the Oculus Reading Probe II, the MNREAD Charts, the SKread Charts, and the RADNER Reading Charts. The test items used for these reading charts differ among the charts and are standardized to various extents. The Bailey–Lovie Charts, MNREAD Charts, SKread Charts, and RADNER Charts are also meant to measure reading speed and allow determination of further reading parameters such as reading acuity, reading speed based on reading acuity, critical print size, reading score, and logMAR/logRAD ratio. Such calibrated reading charts have already provided valuable insights into the reading performance of patients in many research studies. They are available in many languages and thus facilitate international communication about near visual performance. In the present review article, the backgrounds of these modern reading charts are presented, and their different levels of test-item standardization are discussed. Clinical research studies are mentioned, and a discussion about the immoderately high number of reading acuity notations is included. Using the logReading Acuity Determination ([logRAD] = reading acuity equivalent of logMAR) measure for research purposes would give reading acuity its own identity as a standardized reading parameter in ophthalmology.

## Introduction

The near visual properties of our patients, particularly the ability to read, can be affected by many eye diseases. Since the treatment of eye diseases could be significantly improved, and patients who suffer from sight-threatening eye disease share a desire to regain a comfortable reading ability, it is evident that there is increasing clinical interest in well-standardized, calibrated reading charts [1–15]. Accordingly, this review gives an overview of the history and background of modern logarithmically progressing reading charts that can be considered calibrated for the assessment of functional vision [16, 17].

This article is also meant to spark interest in the concept of the necessity for calibrated reading charts in order to achieve international comparability in reading acuity measures, as is already the case for single-optotype distance acuity. Therefore, only those reading charts that can be considered calibrated are discussed here, i.e., those whose standards are in accordance with the standards of the Visual Function Committee of the International Council of Ophthalmology (ICO) [16] and also meet the requirements of the mathematical standards of EN-ISO 8596 [17]. The print sizes of these reading charts were investigated with a measuring microscope in a previous study [18].

Holladay recently indicated that, by analogy to the standards for distance acuity measurements with single optotypes, near-vision measurements must also conform to the same visual angle as distance measurements [19], and he developed a near acuity card using Sloan letters and the EDTRS format. The definition of the relationship between visual angle and optotype size had first been introduced by Snellen in 1862 [20]. It is still the mathematical basis for the construction of optotypes and for all reliable visual acuity notations. However, except for the Birkhaeuser charts, which were produced in 1911 [21] (see below: historical aspects), this relationship has not been applied to the heights of lower-case letters in reading charts for more than a century, most likely because the height of lower-case letters was never a criterion of interest in the printing business. Therefore, the exact height of lower-case letters was not known for hot-lead printing, and until now it could not be determined with the software available for current professional printing. The heights of lower-case letters still have to be determined with a microscope [18, 22]. It must be noted that such measurements come with the risk of artifacts, leading to considerable inaccuracy, and that accurate print sizes below a reading acuity of 0.32 at 40 cm (Snellen: 20/63) are difficult to achieve. Nevertheless, modern printing techniques have allowed us to achieve accurate print sizes with a deviation of no more than 0.01–0.03 of a millimeter, as in, for example, the RADNER Reading Charts. Thus, it is possible to produce reading charts in accord with the desire of clinical professionals to work with measuring tools of the highest accuracy.

It is well accepted that reading words or sentences is a more complex function than is reading single optotypes on an acuity chart [23], because individual letters within words are more difficult to recognize [24, 25]. Accordingly, routine single-optotype distance acuity has been shown to be a limited predictor of reading performance and, thus, cannot elucidate the full functional impairment of several ophthalmic diseases [26, 27]. Reading charts are therefore included as part of an evaluation to ensure a complete evaluation of visual properties. It seems evident that a reading chart standard, by analogy to distance acuity standards, is required in order to allow for comparable measurements of reading parameters, such as reading acuity and speed. In 1988, the Visual Function Committee of the ICO published a standard for reading charts [16], aiming to establish calibrated reading acuity measures. In addition, the mathematical backgrounds of the EN ISO 8596 standard [17] have come to be considered a conceptual requirement for calibrated reading charts.

Only a few reading charts have been designed upon these useful standards or standards equal to these: (a) the Bailey–Lovie Word Reading Chart [28], (b) the Oculus Reading Probe II (Oculus Corporation, DE, USA), (c) the Colenbrander English Continuous Text Near Vision Cards (Precision Vision, Woodstock, IL, USA), (d) the MNREAD Charts [29] (Precision Vision), (e) the SKread Charts [50] (Precision Vision), and (f) the RADNER Reading Charts [30–32] (Neumed AG, AT; Precision Vision). The last four reading charts are available in several languages.

The present review article discusses the backgrounds of the modern logarithmic reading charts that can be considered to be calibrated in accordance to the standards of the ICO and EN ISO 8596.

## Historical aspects of reading charts

In the second half of the nineteenth century, the ophthalmologists Küchler, Jaeger, Donders, Snellen, Green, Landolt, Monoyer, Nieden, Parinaud, and Pflüger developed the current standards for visual acuity measurements. In 1843, Küchler developed distance acuity charts using single words, and in 1854 Jaeger published the “Schrift-Scalen” (Jaeger Charts) [33, 34]. Such developments sparked interest in the idea of the necessity for standardization in visual acuity measurements. Inspired by a formula of Donders (1861), Snellen published the principle of optotype construction in 1862 [20], and in 1867 and 1868, Green introduced the idea of logarithmic progression of optotype sizes [35, 36].

However, similar standards have not been applied to reading charts. Therefore, the historic reading charts, such as the Jaeger [34], Nieden [37], and Parinaud charts, suffer from a considerable lack of standardization (Table 1). Accordingly, their print sizes (letter heights) are not standardized and do not logarithmically progress, most likely because of the limitations of earlier printing techniques.

**Table 1:** Reading acuities measureable with modern and historic reading charts

Modern calibrated reading charts^1^		Parinaud	Jaeger German ∼1995	Jaeger English 1856	Nieden	Birkhaeuser 1911
logRAD logMAR	Decimal 32cm	Decimal 32 cm	Decimal 32 cm	Decimal 32 cm	Decimal 32 cm	Decimal 32 cm
−0.2	1.6	–	–	–	–	1.50
−0.1	1.25	–	–	–	–	1.26
0.0	1.0	–	–	–	–	1.06
0.1	0.8	–	–	–	–	0.93
		P1.5 = 0.72	–	J1 = 0.81	–	0.80
0.2	0.63	–	J1 = 0.63	J2 = 0.66	N1 = 0.61	0.74
				J3 = 0.55	N2 = 0.59	0.63
0.3	0.5	P2 = 0.48	-	J4 = 0.48	N3 = 0.46	0.50
0.4	0.4	P3 = 0.40	J2 = 0.43	J5 = 0.40	N4 =0.40	0.40
			J3 = 0.38	J6 = 0.35	N5 = 0.37	
0.5	0.32	P4 = 0.33	–	J7 = 0.32	–	0.32
0.6	0.25	P5 = 0.29	J4 = 0.27	J8 = 0.30	N6 = 0.29	-
			J5 = 0.25^2^	J9 = 0.27	N7 = 0.27	-
			J6 = 0.25^2^	–	N8 = 0.25	-
		P6 = 0.23	J7 = 0.23	J10 = 0.24		-
0.7	0.2	–	J8 = 0.20		–	0.20
0.8	0.16	P8 = 0.18	J9 = 0.18		N9 = 0.17	-
		P10 = 0.16				-
0.9	0.125	P14 = 0.12				-

^1^ RADNER, MNREAD, Bailey–Lovie, Colenbrander, SKread, Oculus.

^2^ Jg5 and J6 have the same print size but different font type.

An exception to these non-standardized charts is the reading chart developed by Birkhaeuser in 1911 [21, 38]. Birkhaeuser, who was an ophthalmologist and the son of the owner of the Birkhaeuser Printing House, used a photochemical printing technique that allowed him to print logarithmically progressing print sizes of notable accuracy (Table 1). Although he tried to develop a font type for lower-case letters that was in accordance with the principles postulated by Snellen for optotypes [38], he finally abandoned this idea and used an Antiqua typeface that appeared to be closest to the Snellen principle. Interestingly, the typeface he chose is very similar to the Helvetica typeface that has been chosen (in accordance with the same idea) for the RADNER Reading Charts.

With recent printing techniques, it is possible to print letter heights with an accuracy between 0.00 and 0.03 mm [18, 30, 31], whereas the historic reading charts have often been printed only with the limited print sizes available for hot-lead typesetting [22]; this limitation is responsible for the lack of comparability and standardization of the historic reading charts. It is also an explanation for the many different versions of the English Jaeger charts [39], which are barely comparable to each other, and are not at all comparable to the German or other language versions. In addition, during the two world wars, almost all of the original historic reading chart materials were lost and had to be replaced by provisional versions of mostly unknown origin, causing a further worsening of standardization. Unfortunately, these provisional versions have never been questioned and revised.

**Fig. 1:**
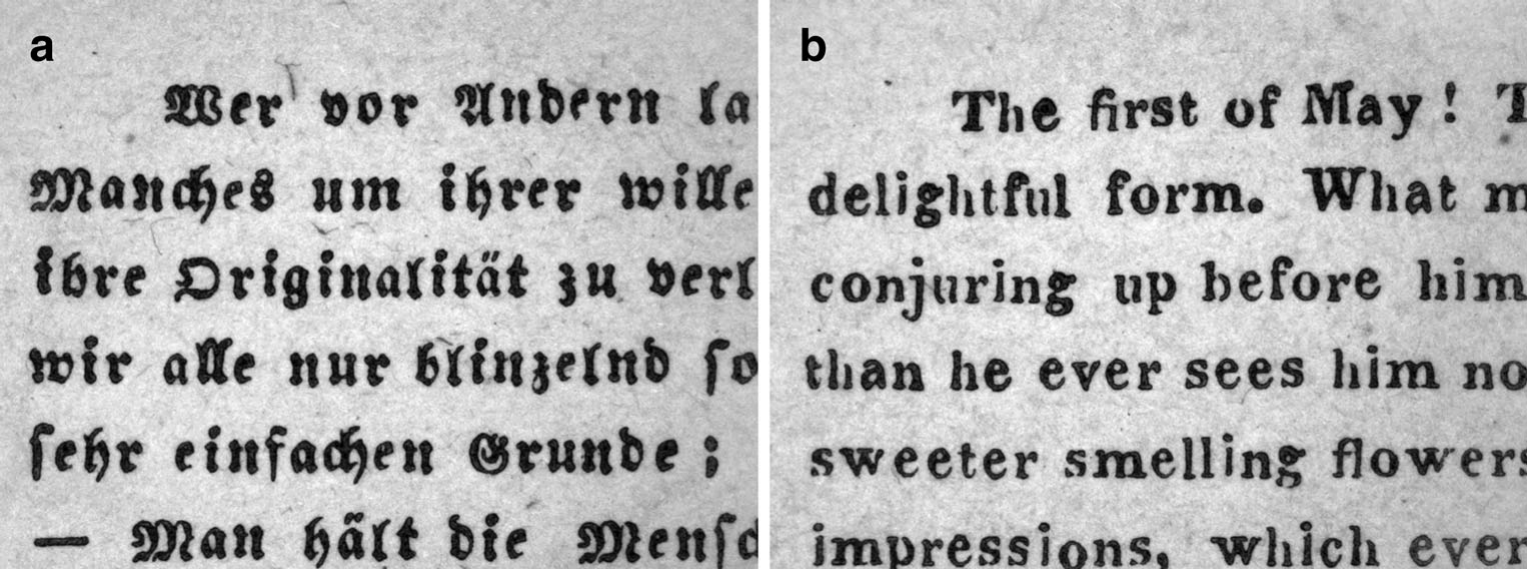
Jaeger Schrift-Scalen 1856: (**a**) German J1 compared with (**b**) the English J1. (**a**) Photographic representation of J1 of the German paragraph and (**b**) J1 of the English paragraph taken from the original Jaeger Schrift-Scalen from 1856 (magnification: 65×). Note that the German version was printed with Gothic letters, while an Antiqua typeface was used for the English version, indicating that even the original version did not represent a comparable international standard

Jaeger’s Schrift-Scalen were developed by the Viennese ophthalmologist Eduard Jaeger von Jaxtthal in 1854 [33, 34]. They represent the first accepted standard before Snellen published his definitions for the standardization of optotypes in 1862 [20]. However, even the original versions did not constitute a comparable international standard because the German version was printed with Gothic letters, whereas an Antiqua typeface was used for the English version (Fig. 1). In the current version of the German Jaeger charts, there are a number of nonconformities with modern requirements for visual acuity tests [22] (Table 1). J5 and J6 have the same print size (1.95 mm in height) and different font types. J1 represents a decimal acuity of 0.63 (Snellen: 20/32) at 32 cm, and J2 corresponds to a decimal acuity of 0.43 (Snellen: 20/47) instead of 0.5 (Snellen: 20/40). In addition, the print sizes of J3 and J4 differ by more than two log units. Similar nonconformities can also be found in the Nieden reading probe [37, 40] and the Parinaud reading charts, two reading charts that are still available [Table 1].

Because historic reading charts such as the Jaeger, Parinaud, and Nieden charts lack useful standards, it seems evident that the evaluation of reading performance using these charts is not suitable for research purposes. Therefore, because calibrated reading charts that are in accordance with recent standards [16, 17] are now available, the historic reading charts should be considered obsolete for the purposes of research and medical documentation of our patients’ reading acuity.

## Modern logarithmic reading charts

Reading has been investigated from many different perspectives [41–46]: e.g., as cognitive, oculomotor, and sensorimotor interactions. Thus, reading tests have become useful investigative tools for several fields of research, including psychology, neurology, and psychiatry. In addition, reading tests are also used for evaluating reading competence [47] and diagnosing reading disabilities such as dyslexia [43]. Another approach has involved the use of reading tests and reading charts in clinical ophthalmology [28–32, 48–53]. However, since the historic reading charts were not standardized at all and could not be used as reliable tools for research purposes (Table 1), the value and potential of standardized reading acuity measures are still underestimated.

About four decades after Birkhaeuser’s reading charts of 1911 [21], the logarithmic progression of the print sizes became again a subject of interest for reading charts. Aiming to overcome the questionable Jaeger standard, Law published in 1951 [54] and 1952 [55] the idea of the N-notation, which is based upon the point (pt) system. However, between N5 and N10, the recommended progression of print sizes is only approximately logarithmic, and between N12 and N48, it definitely is not logarithmic. This approach was followed in the early 1960s by the logarithmic Sloan Reading Cards [49], and then in 1980 by the logarithmic Bailey–Lovie Word Reading Charts [28]. In 1988, the Visual Function Committee of the ICO [16] published standards for reading acuity measurements. These standards stipulate, in short: (a) by analogy to the standards of visual acuity measurements, the print sizes of reading charts have to progress logarithmically, (b) it is desirable that the test conditions, optotypes, and chart design used are calibrated, (c) the test distance has to be specified in all instances, (d) for reading charts, continuous text materials are desirable, and (e) the typeset material should be based upon the distance at which the height of lower-case letters such as “o”, “m”, and “x” subtends five minutes of arc. In addition, the mathematical backgrounds of the EN ISO 8596 standard [17] explain and tighten the conceptual requirements of calibrated reading charts.

All of the modern logarithmic reading charts mentioned in this article (ordered by the year of publication) are in conformity with the standards established by the ICO [16] and are also in accordance with the mathematical backgrounds of EN-ISO 8596 [17].

### The Sloan Reading Cards

In the early 1960s, Sloan developed reading cards in order to determine the required power of reading aids [49]. These cards used continuous text paragraphs of different lengths. The font type used for the cards was a reproduction of that used on a standard typewriter at the time. The smallest print size was 1.0 M, which represents a decimal acuity of 0.4 at a reading distance of 40 cm (1.0 M = the overall dimension of the lower-case letters subtending a visual angle of 5 minutes of arc at a distance of 1 meter). The complete series of print sizes was 1.0 M, 1.5 M, 2.0 M, 2.5 M, 3.0 M, 4.0 M, 5.0 M, 7.0 M, and 10 M, approximately representing a logarithmic progression (3.0 M should be 3.2 M and 7.0 M should be 6.3 M; 8.0 M is missing).

### The Bailey-Lovie Word Reading Charts

**Fig. 2:**
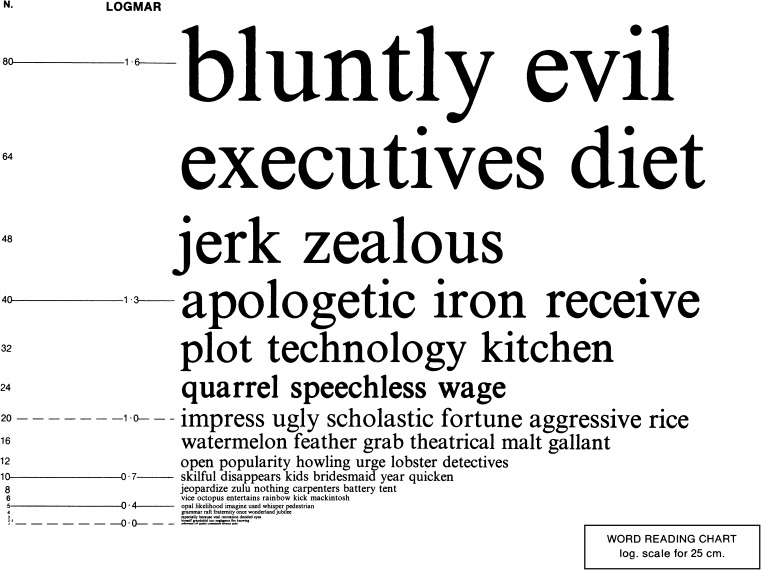
Bailey–Lovie Word Reading Chart : example of one of the Bailey–Lovie Word Reading Charts (original size: 26.0 cm × 20.5 cm}. Printed with the permission of Ian Bailey

In 1980, Bailey and Lovie developed the Bailey–Lovie Word Reading Charts (Fig. 2), which were designed to determine reading acuity and speed in one simultaneous examination with a reading chart [28]; this principle has also been applied to the MNREAD [29] and RADNER Reading Charts [30–32]. Bailey and Lovie designed a word-reading chart with a logarithmic size progression and used unrelated words. Following the recommendations of the British Faculty of Ophthalmologists, [54, 55], they used the Times Roman typeface. They further decided to use four-, seven-, and ten-letter words at each size level, based on the observation that in patients with age-related macular degeneration (AMD), the word length can affect the readability (some patients prefer longer words, others shorter ones). The words and word order were selected with the intention of having the first letters of the words evenly distributed over the whole alphabet. The frequency of word use also became a selection criterion, and care was taken to avoid obvious syntactic associations between adjacent words [23, 28]. On the charts, print sizes were labeled in N-notation (points), M-units, VAR, and logMAR values given for 25 cm.

### The MNREAD Charts

**Fig. 3:**
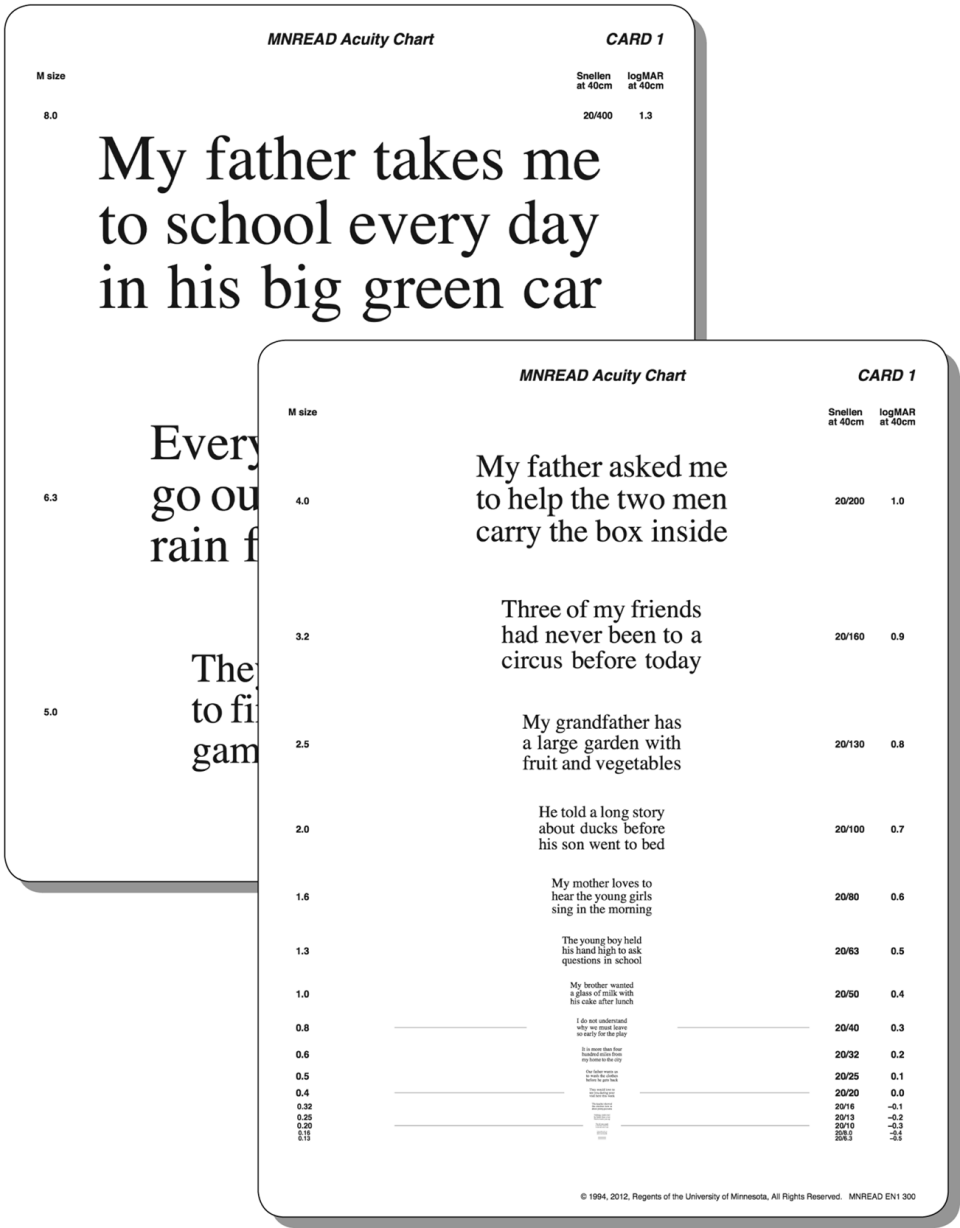
MNREAD Chart (original size: 46.0 cm x 30.0 cm). Printed with the permission of Gordon Legge

Legge and colleagues [51] were the first to use single sentences for a computer-aided test of reading speed, first called the Minnesota low-vision reading test. In this test, sentences were presented to low-vision patients on a computer screen. The print size was very large (6° characters), exceeding the acuity limit of most patients with low vision. In a series of trials, the presentation time for the sentences was reduced until the patient could not complete reading the sentence. The reading speed was then calculated from the number of words read within this last time-period. Subsequently, a card version [52] and then a chart version (Fig. 3) were developed using short sentences over a wide range of print sizes, called the MNREAD test [29]. This test incorporated the concept of “standard-length word” introduced by Carver [56, 57]. The sentences of the MNREAD tests are characterized by their length, which was initially defined as 52 characters including spaces (four lines per sentence) [51, 52], and then for the MNREAD Charts, it was defined as 60 characters including spaces, with an implied period at the end of a sentence (three lines per sentence) [29]. Based on a study by Carver [56], this length turned out to be convenient for scoring reading errors and reading speed when a “standard-length word” is defined to have six characters. In this case, a 60-character sentence consists of ten standard-length words. Using standard-length words helps minimize the variations in scoring that occur as the result of the different word lengths found in different sentences [29, 58]. The MNREAD charts are available in several languages and give the logMAR notation, the Snellen notation, and M-units for 40 cm.

Similar to the test–retest reliability analysis performed for the RADNER Reading Charts [32, 59], a Bland–Altman test–retest analysis (test–retest interval: the same day) was performed in visually impaired patients for the two MNREAD Charts by Subramanian et al. in 2009 [60]. Virgili published the coefficient of repeatability obtained from a group of children with the Italian MNREAD Charts. The studies showed good repeatability in visually impaired adults and children [61].

### The RADNER Reading Charts

**Fig. 4:**
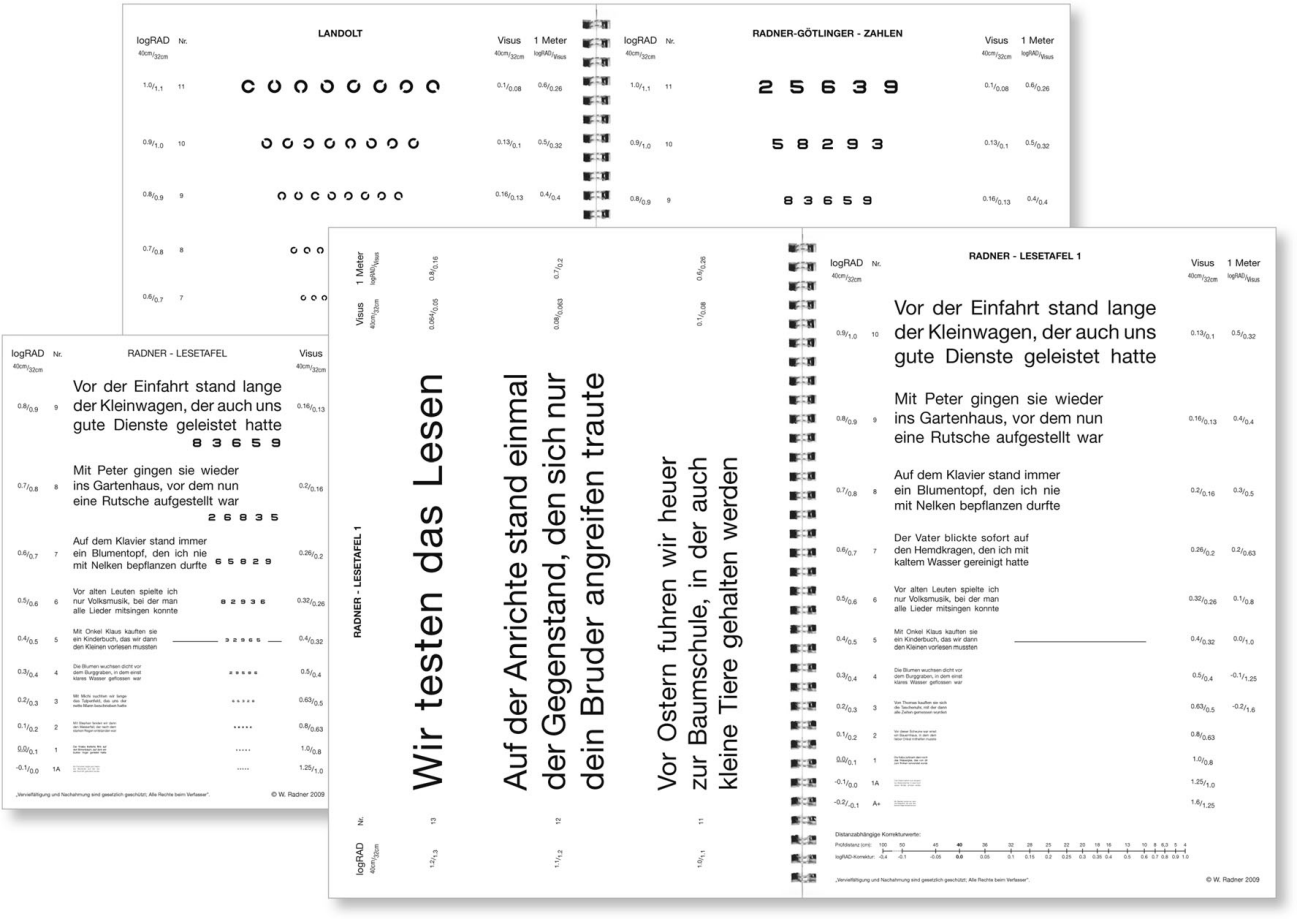
RADNER Reading Charts: Radner Reading Charts, as exemplified by the German version. Four text reading charts, a page with Landolt rings, and a page with numbers are provided in the booklet (original size: big issue, DIN A4 29.7 cm × 21.0 cm; small issue, DIN A5 21 cm × 14.8 xm)

Since the statistical definition of test items is an inevitable requirement for a medical test used in patient care, the aim in developing the RADNER Reading Charts (Fig. 4) was to achieve best accordance with optotype standardization [16, 17, 20]. For these charts, the concept of “sentence optotypes” is essential and was introduced in order to provide clear definitions for the test items, stop criterion, difficulty, and reading length, and to keep the geometric proportions between the test items as constant as possible [30, 31].

#### The concept of sentence optotypes

A series of test sentences was generated (main clause followed by a relative clause), all of which had to be as comparable as possible in terms of the number of words (14 words), word length, number of syllables per word, position of words, number of characters, lexical difficulty, and linguistic aspects such as grammar and syntax [30, 31]. These sentence optotypes (Fig. 4) of three lines and 14 words (main clause followed by a relative clause) incorporated 82–84 characters, including spaces (27–28 characters per line) and 22–24 syllables. The position and length of the words was defined by specified rules [30, 31]; for example, the first line (five words) starts with a word of three letters and one syllable, followed by a noun with two syllables in position two or three. The second line also starts with a word of three letters and one syllable, which is followed by a noun of ten letters and three syllables. Then the relative clause starts with three short one-syllable words and so on [30, 31]. By testing a group of 198 volunteers, the most equivalent sentences optotypes were statistically selected with respect to reading length and difficulty by introducing a narrow “reading length interval” [30, 31]. Finally, 38 sentence optotypes were statistically selected. The Cronbach’s alpha and the corrected item total correlation were well above statistically required limits [30, 31, 62]. The reading speed correlated well with that obtained for long paragraphs, indicating the high validity of these test items.

#### Standardization of the reading charts

For standardizing the RADNER Reading Charts, a methodical design, including Bland–Altman plots for reading chart standardization, was established in order to investigate the test–retest reliability and interchart reliability and to evaluate a reading chart through a variance component analysis [32, 59] for the German and the Dutch versions. The results demonstrated that these reading charts provide highly reproducible measurements of reading acuity and speed in individuals with no, moderate, or increased visual impairment (test–retest interval: 3 to 4 weeks; Latin square design). In addition, they have shown that the reading charts provide reliable, reproducible, and comparable measurements of reading performance for research and clinical practice.

A sans-serif Helvetica typeface was used for the reading charts. All notations (decimal, Snellen, M-units, and logRAD) are given for 40 cm and 32 cm (in the German version also for 1 meter). Except for logRAD, which is given in all language versions, the notations shown on the charts depend on the tradition of reading acuity determinations of the countries in which the particular language is spoken. A logRAD adjustment scale for different reading distances is provided on every chart (range: 4 cm to 50 cm). In addition, a page with numbers and one with Landolt rings are included in the booklet.

The concept of sentence optotypes has been applied to 12 different languages (a total of 1,323 volunteers have been tested in order to standardize the sentence optotypes in the 12 languages). The Radner Reading Charts are available in German, Spanish, English, French, Dutch, Italian, Swedish, Danish, Portuguese, Turkish, Hungarian, and Romanian, and further languages are in progress.

### The Colenbrander Continuous Text Near Vision Cards

**Fig. 5:**
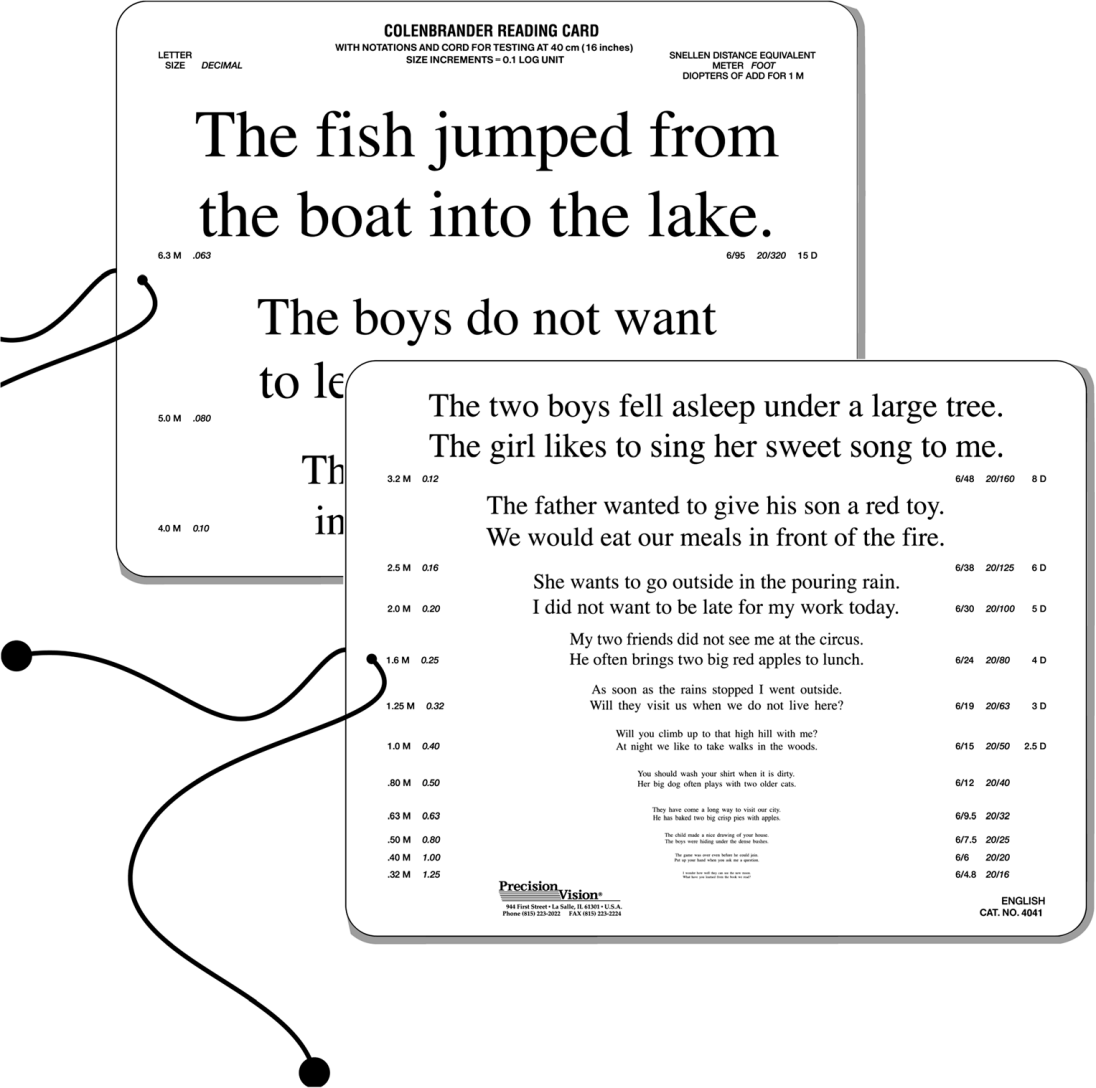
The Colenbrander English Continuous Text Near Vision Cards (Original size: 23.0 cm x 18.0 cm) Printed with the permission of August Colenbrander

The Colenbrander Continuous Text Near Vision Cards (Precision Vision, Woodstock, IL, USA; Fig. 5) are also logarithmically scaled; they are available in 12 languages. For use at 40 cm, they cover decimal acuities from 0.063 to 1.25 and also give the Snellen notation and M-units; logMAR notation is not given. To maintain the correct reading distance, a 40-cm cord is mounted on the cards, and for use in low vision, the cards come with a ruler to facilitate use at shorter distances for lower acuity levels. The test sentences have 44 characters including spaces and a different number of words (nine to 11 words). For decimal acuities from 0.063 to 0.1, one sentence is presented per print size, and for 0.12 and smaller, two sentences are presented. These reading cards are also available as mixed-contrast cards on which high and low contrast (20% Weber) are presented side-by-side on the same card.

### The Smith–Kettlewell Reading Test (SKread)

**Fig. 6:**
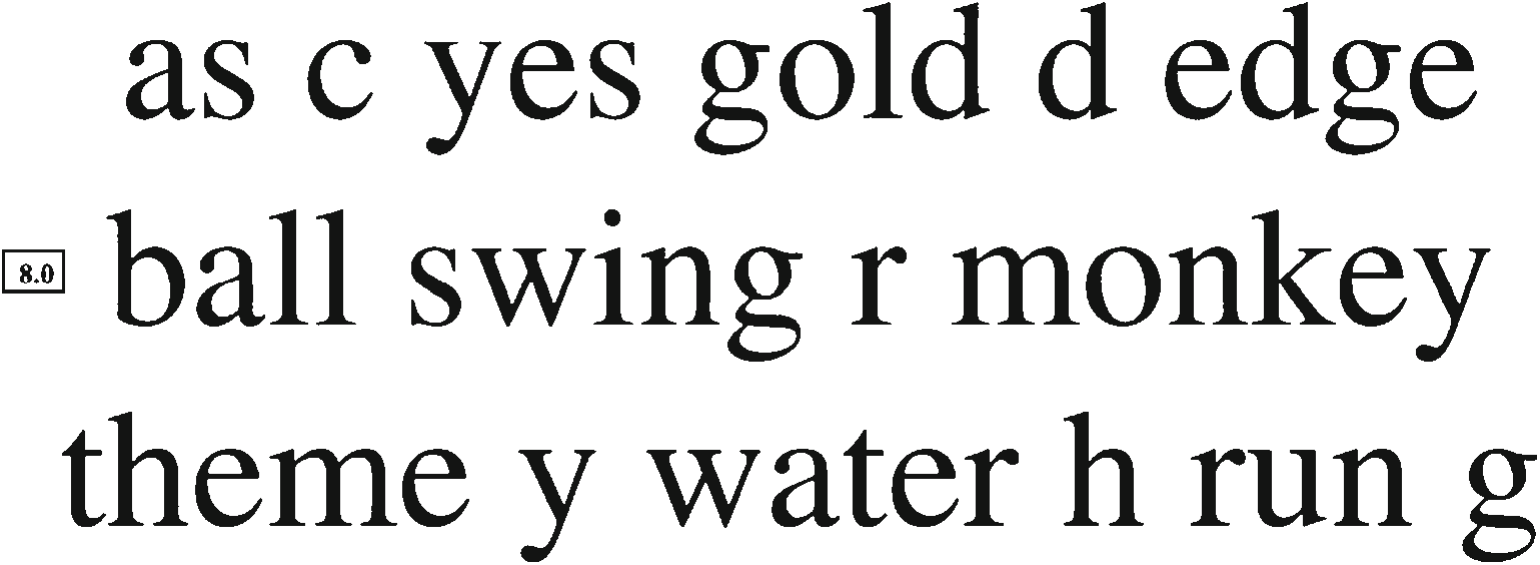
SKread paragraph: Example of a paragraph of the SKread Charts. Unrelated words are interrupted by single letters. Printed with the permission of Manfred MacKeben

The SKread Test (Precision Vision, Woodstock, IL, USA) was developed to assess the reading performance of low-vision patients and simultaneously allow estimation of the location of scotomas [50]. It can also be used to determine the magnification needs of such patients. Each test paragraph contains six single letters and ten unrelated, randomly chosen words (60 characters including spaces; 47 letters, Fig. 6). The number of words with two, three, four, five, and six letters is equal in all paragraphs. Words that can stand alone with letters missing from the beginning or the end of the word were especially included. This test principle was chosen because the authors wanted performance to depend upon word and letter recognition alone, and wanted to exclude linguistic aspects such as grammar and syntax. Print sizes progress logarithmically, and are labeled in M-units from 0.4 M to 4.0 M. No other notation is given.

### The Oculus Reading Probe II

**Fig. 7:**
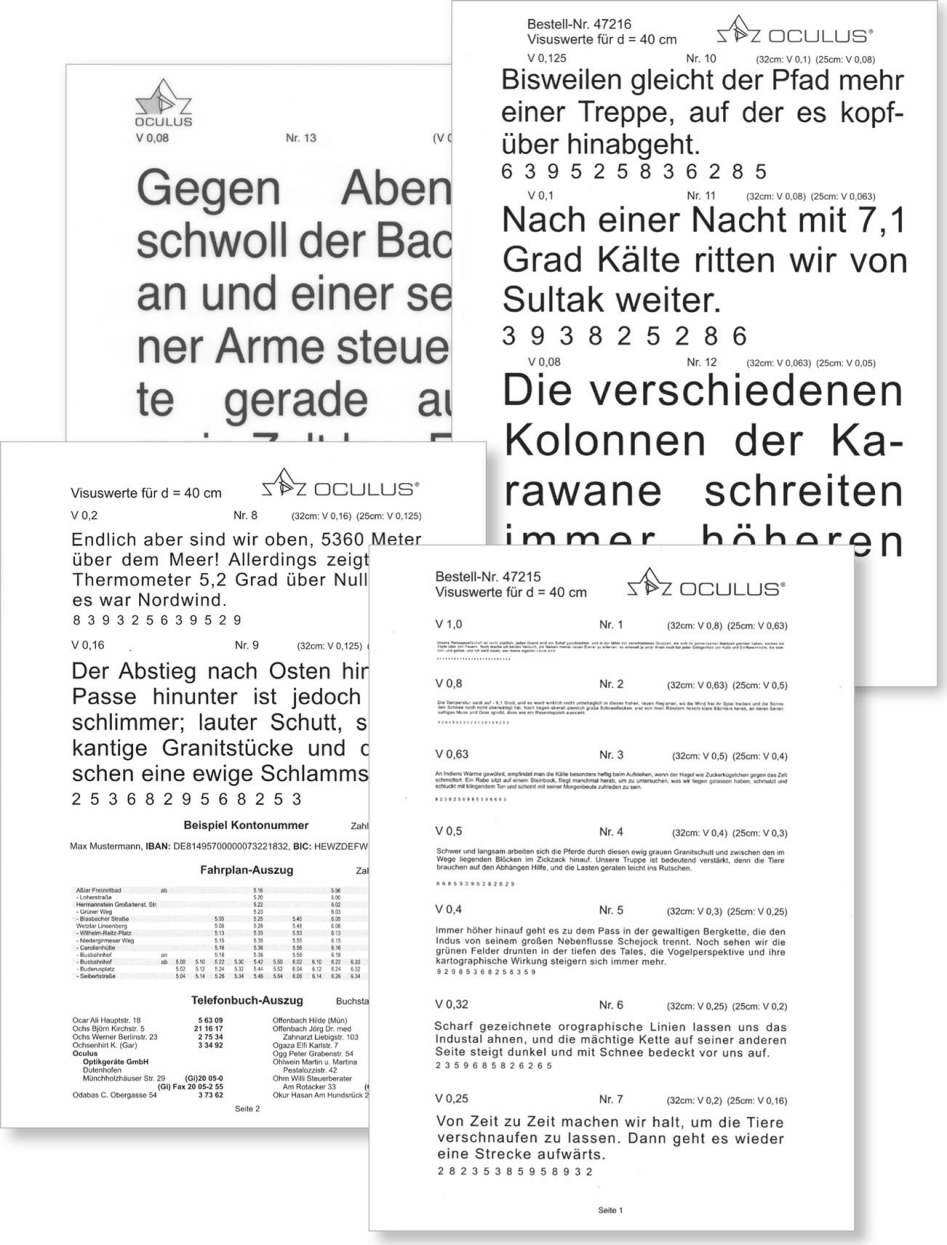
Oculus Reading Probe: (Original size: 21.0 cm × 14.8 cm) Printed with the permission of the Oculus Corporation

The Oculus Reading Probe II (Fig. 7) uses long paragraphs from a book written by Sven Hegin and from *The Jungle Book* by Rudyard Kipling. The print sizes increase logarithmically from decimal acuity 1.0 to 0.04. Reading acuity is given for 25 cm, 32 cm, and 40 cm. Within the booklet a timetable of train schedules, an example of a telephone book, and SEPA numbers are also given. Music, Landolt rings, and tumbling Es are also provided.

The Oculus Reading Probe II is available in German, and is an innovative example of calibrating an already well-recognized reading chart with modern standards. In 2015, the OCULUS Corporation reissued their reading charts. They asked the author of this article to collaborate in conforming the print sizes of the Oculus reading probe to those of the RADNER Reading Chart (Fig. 4), which are in accordance with the standards of the ICO committee [16] and the EN ISO 8596 [17] (the author was responsible for the accuracy of the print sizes; measurement system: ultra-measurement-lograd©). Now, the two leading reading charts in German-speaking countries provide calibrated reading acuity measures. This was the first time that two different reading chart systems had been calibrated so that the print sizes, and therefore the reading acuity measures, were equalized.

### Other ophthalmic reading tests

The present review article is focused on standardized reading charts for measuring reading acuity and speed, and therefore on aspects of standardized print size, print-size progression, and test-item definition. Such reading charts are meant to achieve an international standard for reading acuity measures and permit standardized investigations of further aspects of reading performance. Nevertheless, other reading tests that cannot be considered calibrated still deserve a brief mention.

#### Eschenbach and Zeiss reading tests

The Eschenbach and Zeiss reading tests use long paragraphs and also provide a logarithmic progression of the print sizes. These reading tests are thought to determine the magnification needs of low vision patients. No visual acuity notation is given. The test distance is 25 cm, and the print sizes range between a decimal acuity of 0.2 to 0016 (Eschenbach) and 0.2 to 0.001 (Zeiss).

#### Keeler Reading Test Types

The Keeler Reading Test Types use long paragraphs. The print sizes range from N5 cm to N48, and do not progress logarithmically. The smallest print size is N5. For N5, the lower-case letter height of the typeface used was found to be 0.973 mm, representing a decimal acuity of only 0.60 at 40 cm. A recommended reading distance is not provided. Between N5 and N10, logarithmic scaling is almost, but not completely accurately, achieved. From N12 (the log-scale would require N12.6) to N 48, the progression of N-sizes cannot be considered logarithmic, since, for example, N14 should be N15.8, and N36 should be N31.5.

#### IReST

The IReST (Precision Vision, Woodstock, IL, USA) [53] is a low-vision reading test and not a reading chart. It comes as a booklet, and uses long paragraphs for analyzing speed and fluency of reading in low-vision patients. Ten long paragraphs with different word counts have been developed for each of the 17 languages. By testing 25 normally sighted subjects (36 for Japan), the mean reading speed ±SD is calculated for each paragraph and is given, together with the word count, next to each paragraph. There is evidence that significant differences can occur between paragraphs [63].

#### Radner paragraph optotypes

Recently, a more elaborate concept for the standardization of long paragraphs (paragraph optotypes) used for reading charts and reading speed analysis has been published [56]. Seven long paragraphs were developed, each consisting of 111 words, 179 syllables, and 660 characters (710, including spaces). These paragraphs were also constructed so that words with the same number of syllables were in exactly the same position in the text in all paragraphs. Statistical analysis showed good reliability and validity for these paragraphs. However, it was found that a statistically significant difference in reading speed could appear between long paragraphs, even when the construction of the paragraphs was highly equivalent. Ultimately, two sequences of three paragraphs each, as well as eight of 21 pairs of paragraphs, were statistically selected for which the reading speed was not significantly different.

## Reading parameters

**Fig. 8:**
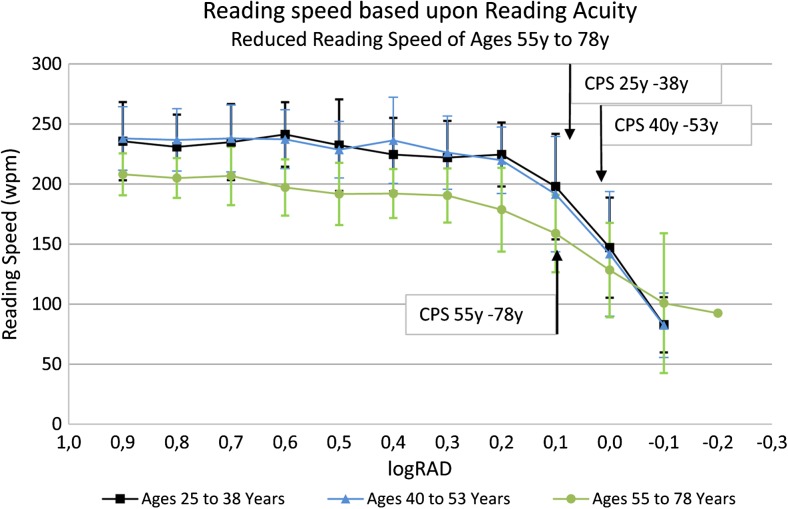
Reading speed based upon reading acuity: The figure shows the mean reading speed based upon reading acuity and the mean critical print size for three different age groups. Note the difference in the mean reading speed between the two groups of ages 25 to 38 years and 40 to 53 years and the group of older readers aged 55 to 78 years

**Fig. 9:**
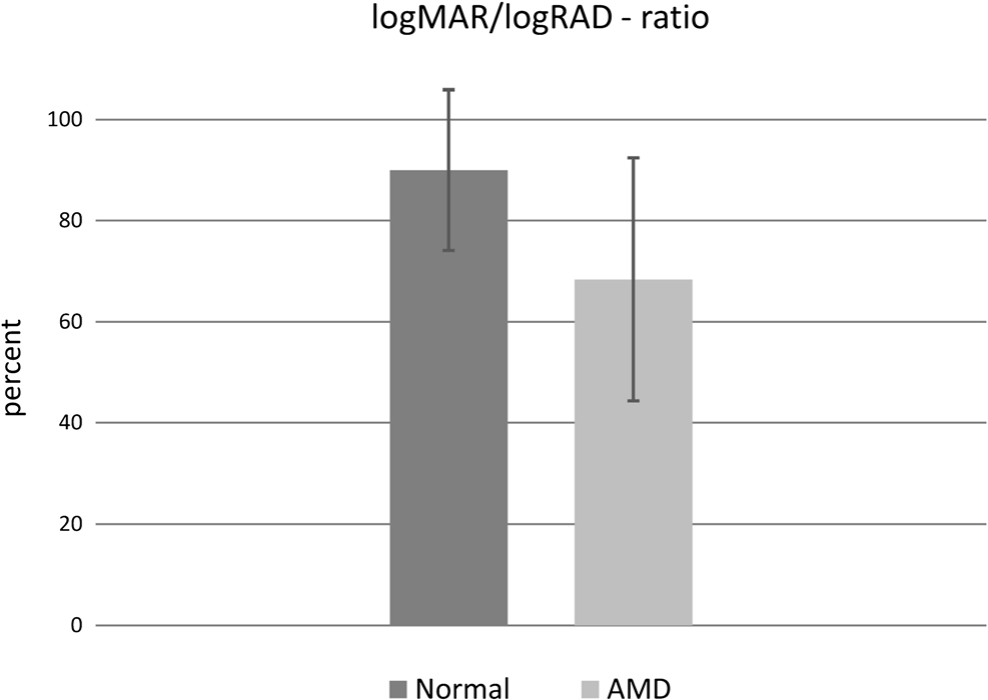
logMAR/logRAD ratio: the logMAR/logRAD ratio shows the reading acuity (logRAD) as a percentage of the distance acuity (logMAR). In this figure, it is exemplified by the logMAR/logRAD ratio of a normally sighted person with healthy eyes and that of patients suffering from AMD. The logMAR/logRAD ratio is considerably lower in AMD patients

**Fig. 10:**
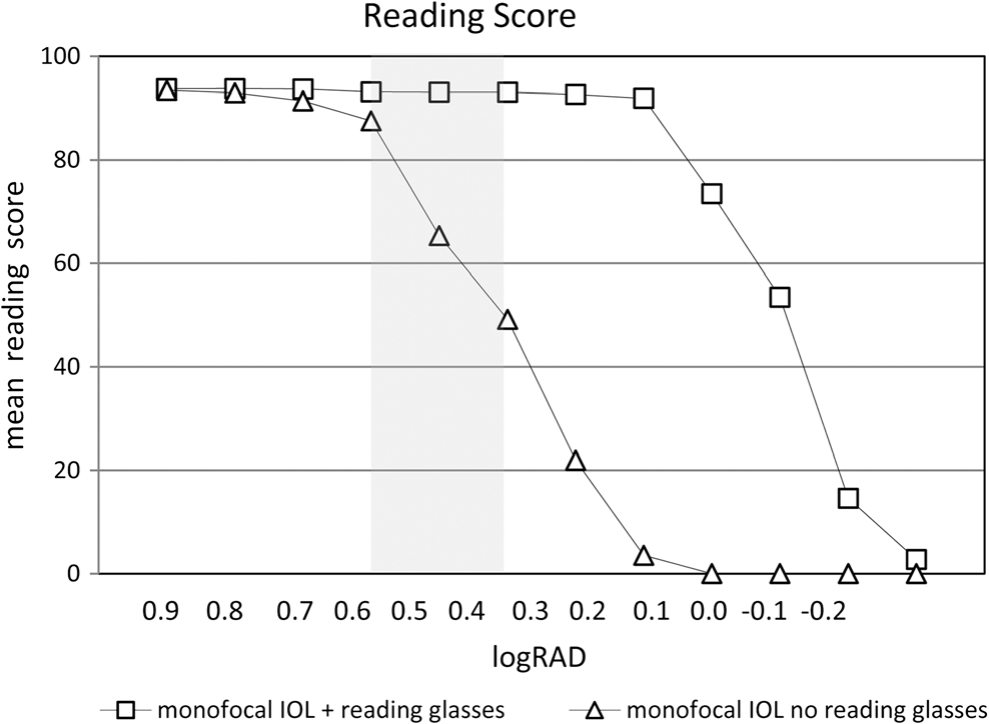
Reading score: the figure exemplifies the reading score per print size obtained from patients with monofocal IOLs reading binocularly, either with best corrected reading acuity or without reading glasses (20 patients were investigated). Although some of the patients could read newspaper-sized print without reading glasses under good light conditions, their reading performance was significantly reduced. The *gray area* indicates the range of print sizes, from newspaper (*left edge*) to high-gloss journals (*right edge*)

In addition to reading acuity, the reading acuity score, the maximum reading speed, and the mean reading speed, several other reading parameters can be analyzed, such as the reading speed based upon reading acuity (Fig. 8) and the logMAR/logRAD ratio (Fig. 9). The reading score [10] (Fig.10) which was developed to compare the reading acuity based upon reading speed obtained under different reading conditions, can also provide useful information about functional vision.

An interesting parameter of clinical value is the critical print size (CPS) [29–32, 60, 64]. The CPS can either be defined by the examiner as the smallest print size that was read with normal reading speed or, as given by Subramanian et al. [60], “the smallest print that supports the maximum reading speed and is identified based on the criterion that all the following (smaller) sentences are read at a speed that is 1.96 times the standard deviation below the average of the largest preceding sentences.” However, the variant component analysis for the examiner-based CPS determination [32] revealed that the patients accounted for only 31% to 54% of the entire variance, whereas for reading acuity, the patients accounted for 85% to 94% of the whole variance (the higher this percentage, the more likely it is that the test is dependent on the person’s reading ability). In comparison to the other variables, the variance component analyses revealed that, for the CPS, a considerable proportion of the variability came from unidentified sources. One explanation for this difference might be that the CPS is not a measurement in the same way as the reading acuity or speed, since it has to be set by the examiner at the smallest print size the patient can read with normal reading speed [32]. For the statistical definition of the CPS [60], it was also found that the coefficient of repeatability was considerably weaker than that for reading acuity and reading speed. In 2011, Patel et al. [65] reported even weaker coefficients of repeatability for the CPS obtained with the MNREAD Charts, and using different methods to determine the CPS did not lead to an improvement in the coefficient of repeatability of the CPS. In the author’s opinion, the weak statistical repeatability is a result of limited flexibility, because the CPS is strictly given in full 0.1 log units [60]. However, the reading speed at the CPS varies considerably between patients, and it is likely that the real CPS would be somewhere between log units (the closer the reading acuity is to the CPS, the faster reading speed will be). Such variations are not represented within the CPS values. Nevertheless, the CPS provides valuable information for clinical purposes.

## Stop criteria

Reading charts using single sentences, sentence optotypes, or sequences of unrelated words also permit the introduction of a stop criterion (the length of time that the subject is allowed to read before that individual trial is stopped). This criterion can be freely chosen with regard to the requirements of clinical routine or a particular study design.

For the RADNER Reading Charts we suggest a stop criterion of 20 seconds [30, 31]. This corresponds to a reading speed of about 40 wpm (reading speeds of 40 wpm or lower suggest the reading of single words, i.e., spot reading [41]). The lower limit for fluent, sense-capturing reading has been found to be at about 80 wpm [41]. However, although it is of interest to know how many patients of a study group read faster than 80 wpm, using 80 wpm for a stop criterion does not seem to be acceptable: 80 wpm represents a reading time of just about 7 seconds per sentence for the MNREAD Charts and 10 seconds per sentence for the RADNER Reading Charts. In normal-sighted persons, these speeds per sentence are too close to the reading speeds at the CPS. Using this limit, the patient’s full visual potential (i.e., best reading acuity) cannot be shown. However, best reading acuity is a result that is as important as is the best distance acuity. Thus, reading acuity should be determined by procedures analogous to those used for single-optotype distance acuity [16, 17, 19, 20]. Accordingly, stop criteria have to be chosen in a way that guarantees the ability to deduce information about the best reading acuity.

## Notation

In 1874 Snellen and Landolt mentioned in their chapter [35]: “*It is regrettable, that for the determination of visual acuity the consistency of scaling has not been more considered. Whereas the world vigorously pursues a uniformity for mintage, weight and measure of length, it seems to be the other way around in our field, trying to make the notations as diversified as possible*.” Just a few years later, logarithmic progression and the decimal notation as well as the Snellen notation became the accepted standards in clinical routine worldwide. For reading charts, however, it seems that the statement of Snellen and Landolt is still true. Jaeger, Nieden, Parinaud, Decimal, Snellen, M-size, the N notation, line numbers, logMAR, logRAD, and VAR notations are currently in use, depending on the users’ location and educational background. It is not within the bounds of this review to present and discuss all of this notation in detail. However, to mention a few types:

### M-Size

Sloan introduced the M-unit notation [66]. The M-unit is the letter height that corresponds to a visual angle of 5 min of arc at a distance of 1 meter. The other print sizes derive from upwards and downwards multiplication, with decimal logarithmic steps of 10^0.1^ starting from 1.0 for 1 meter. The notation is given in terms of the factor used to modify the print size, multiplying it by the letter height at 1 meter (=1 M-unit). An advantage of the M-unit notation is that is equal to the distance in meters at which a letter is seen under 5 min of arc (analogous to a decimal acuity of 1.0, or Snellen 20/20). Although it implies a relationship to the print size, a disadvantage of the M-unit notation is that from the point of view of users of the decimal system, it is upside-down and is not calculated using the real test distance used. The M-unit notation is related to a fixed test distance of 1 meter but is also used for 40 cm and other reading distances. Another disadvantage is that for statistical analyses −logM has to be used.

### N-Notation

In 1951, Law published, on behalf of the British Faculty of Ophthalmology, a recommendation for a reading type standard [54, 55]. One of his main reasons for doing so was to replace the Jaeger notation, which was perceived to be obsolete. He recommended the use of Times Roman typeface, standard spacing, and a notation that is based upon the point (pt) system. Accordingly, the N-notation represents print sizes based upon the point (pt) system, as used in the printing business. However, points represent the height of the block, and not the height of the letter that is mounted on the block. Thus, the letter height can differ considerably between font types while the height of the block remains the same, an unfortunate circumstance for the standardization of print sizes, since the letter height of 10 pt Arial is 1.96 mm, representing a decimal acuity of 0.30 at a reading distance of 40 cm, whereas the letter height of 10 pt Times Roman is 1.69 mm, corresponding to a decimal acuity of 0.34. The difference is almost half a log-unit. In addition, until 1951, many different variations of the Times Roman typeface with different letter heights appeared on the typeface market; today, however, modern printing techniques allow the production of highly accurate lower-case letter heights.

At first sight, a point-based notation seems to be convenient because it is a familiar system that is also used in everyday life. However, it is disadvantageous when a logarithmic progression of print sizes is desirable. Also, the original aim of the N-notation, i.e., to replace the obsolete Jaeger measures, has meanwhile been achieved by the ICO standards of 1988 [16] and by modern reading charts that also consider EN-ISO 8596 [17]; therefore, it could be considered reasonable to rethink the value of the N-notation.

### Snellen fraction

The Snellen fraction expresses the relationship between the test distance (feet or meters) and the distance at which an optotype subtends 5 min of arc. Bailey and Lovie-Kitchin pointed out that when Snellen notation is used for reading acuity, one would have to use the Snellen fraction 0.4/0.4 for a test distance of 0.4 meters (40 cm), and not 6/6 or 20/20 [23]. Nevertheless, Snellen fractions are commonly used, and with 6/6 (meter) or 20/20 (feet) as a reading equivalent (lower-case letter size seen under 5 min of arc at the reading distance chosen), they are likely to be well understood when clear information is provided to explain that they are being used for near or reading acuity.

### Decimal notation (Visus)

The decimal acuity notation is the reciprocal proportion of the visual angle that is calculated from the test distance and the optoype or letter height. It therefore reveals correct and logarithmically progressing values for whatever distance is calculated. It was chosen to produce higher values with better vision and lower values when the vision decreases. The starting point is 1.0 and corresponds to the Snellen principle of optotype construction (the optotype seen at the test distance under a visual angle of five min of arc).

### LogRAD

Since from a psychophysical point of view, reading acuity involves a different visual task than does single-optotype distance acuity, the suggestion was made to use different definitions for the different tasks. This concept led to the introduction of the term log-Reading Acuity Determination (logRAD) for reading acuity measures, the reading equivalent of logMAR [1, 30–32]. The use of logRAD was found to be convenient because it avoids the confusion between distance and reading acuity that is likely to occur when logMAR is used for both distance and reading acuity. In addition, this differentiation of distance and reading acuity follows the principle that different definitions should be used for different functional properties, as is the case for terms in physics used in everyday life (e.g., Hz, Watt, kg, Kp, meter, seconds). Therefore, it seems to be useful to use different terms for distance acuity (logMAR) and reading acuity (logRAD). Use of logRAD would give reading acuity its own research identity.

## Clinical aspects of calibrated reading charts

Clinical outcome studies using calibrated reading charts began appearing in 2002 [1], when the reading performance obtained with a diffractive multifocal IOL was compared to that of a refractive IOL with the RADNER Reading Charts. Since then, a number of studies performed with these standardized logarithmic reading charts have shown that it is possible to obtain detailed information about the reading performance achieved with bi- and multifocal IOLs [1–9], monofocal IOLs [9, 10], or following LASIK/LASEK [11] or refractive laser treatment for presbyopia [12–14]. In addition, the reading performance of patients with different types of cataracts [67] has been analyzed, and the potential for using such reading charts to discriminate among visual impairments caused by cataracts and age-related maculopathy has also been demonstrated [68]. Interesting insights into the reading performance of cataract patients and about potential acuity measurements have also been obtained with the Bailey–Lovie Word Reading Charts [15]. Patients who underwent cataract surgery have also been investigated with the MNREAD Charts: for example, with two types of accommodating IOLs [24] or with regard to the reading performance of patients of working age with diffractive multifocal IOLs [69]. The RADNER Reading Charts have further been used to investigate the reading performance of patients suffering from many diseases, including AMD [70–72], amblyopia [73, 74], infantile nystagmus [75], uveitis [76], treatment of diabetic macular edema [77], macular hole surgery [78], and telangiectasia type 2 [79], as well as that of patients who have undergone various surgical treatments [80–83]. These reading charts have also been shown to be feasible for investigating low-vision patients [63, 84, 85] and have provided insights into the correlation between scotoma size and reading performance [86].

With the MNREAD Charts, patients with retinitis pigmentosa [87], AMD [88, 89], macular pucker and macular hole surgery [90], diabetic macular edema [91], and albinism [92] have been investigated, and further studies about the reading performance of low-vision patients have been performed [93–98]. In one study, the reading acuity was examined with the Bailey–Lovie Word Reading Cards, and reading speed was investigated with the MNREAD test [89].

In a study presenting a new way of standardizing long paragraphs as a functional vision test, it was shown that the reading speed in normally sighted persons changes with age, in terms of reading both long paragraphs and sentence optotypes [48]. The group aged 55 years or greater (mean: 62.90 ± 7.36 years) read significantly more slowly than did the groups aged 20 to 35 years (mean: 26.60 ± 3.72 years) or 36 to 51 years (44.25 ± 4.76 years). This observation that reading performance changes with age was recently confirmed in a retrospective analysis of data obtained with the MNREAD Charts [99]. In that analysis, a break-point for decreasing reading speed was detected at age 40. However, no significant difference was found between the groups aged 20 to 35 and 36 to 51 [48]. A possible explanation for this difference could be related to the retrospectively obtained data that were used: The participants in the study of Calabrese et al. [99] who served as the controls in the previous studies merely read with their “habitual” near refractive corrections [99] and did not read with their best-corrected near vision, evaluated directly before the examination.

Since 2002, a considerable range of clinical studies has shown that calibrated reading charts allow standardized and comparable analysis of reading performance and, thus, of an important aspect of functional vision. Results obtained with calibrated reading charts allow comparison of research studies and are more accurate than less standardized charts in terms of comparing clinical outcomes at different stages of follow-up.

## Discussion

Bailey and Lovie–Kitchin concluded that “reading of words or sentences is clearly a more complex function than is reading the widely spaced letters of a distance acuity chart” [28]. They further stated that, as “compared to isolated letters, the individual letters within words are more difficult to recognize because of interactions with closely packed neighboring letters” [25, 100]; the more important element in reading was found by Bouma to be the recognition of letter and word sequences [101, 102]. It therefore is not surprising that routine single-optotype visual acuity tests have been shown to be poor predictors of reading performance and, thus, cannot elucidate the full functional impairment of many ophthalmic diseases [26, 27, 70].

Accordingly, the appearance of calibrated reading charts has initiated an increasing interest in a standardized investigation of reading performance in patients with visual function from normal to low vision. Reading parameters such as reading speed evaluation based upon reading acuity, the reading acuity score, the critical print size, and the mean and maximum reading speeds have provided interesting insights into the near functional performance of patients prior to and following therapy [1–15, 79–83, 90].

For medical tests used in patient care, substantial statistical analyses of test items are conventionally required. Thus, it is evident that such analyses using adequate statistical methods should also be applied to test items used for reading charts. Different variants of test items have been chosen for reading charts: (a) long paragraphs (Jaeger, Nieden, Oculus, Eschenbach, Zeiss), (b) unrelated words (Bailey–Lovie, SKread), (c) single sentences (MNREAD), and (d) so-called “sentence optotypes” that represent single sentences of main clause followed by a relative clause construction (RADNER). However, statistical parameters have been analyzed and published in detail only for the sentence optotypes of the RADNER Reading Charts [30, 31]. Other test items are defined by the number of characters and/or by the word length [28, 29, 50]. For the RADNER Reading Charts, the aim was to control linguistic aspects by statistically selecting the test items (sentence optotypes), which had been developed to be grammatically equal, using words of equal or similar length in the same positions in the sentences [30–32]. Particular care was taken to avoid anticipation of the sentence’s content that could artificially increase the reading fluency [30, 31, 103]. By introducing tight limits on reading length and the number of errors, inclusion criteria were established [30, 31]: To be selected, the mean reading speed and error score of a sentence optotype had to be within these limits. Furthermore, the Cronbach’s alpha, corrected item total correlation, and inter-item correlations were investigated and found to be well above statistically required limits [30, 31, 62].

However, statistical test-item definitions such as the reading length interval, the Cronbach’s alpha, or the corrected item total correlation are not available for the other calibrated reading charts. For the MNREAD Charts [29], the principle of standard word length as proposed by Carver [57, 58] was adopted in order to achieve comparability between sentences with different numbers of words. Ahn and Legge [104] validated the computerized MNREAD test in low-vision patients by comparing the reading speed obtained with single sentences presented on a computer screen to those obtained from the same patients when they read long paragraphs with their “preferred” magnifiers. Ahn and Legge found that the MNREAD score is a good predictor of magnifier-aided reading speed, and that distance visual acuity is not. In a further study, Ahn et al. [52] presented a printed card version using the same set of sentences and display format. In order to find the simplest method of test presentation, they compared three different methods (hand-held; mounted on a board; inserted into a self-supporting stand) to each other in 23 low-vision patients. No significant differences were found among the three methods. However, the sentences used in these studies consisted of four lines and 52 characters/spaces (13 character/spaces per line) and were different in length from those used for the MNREAD Charts (three lines and 60 characters/spaces) [29].

Another approach to reducing linguistic concerns such as grammar and syntax has been used in the Bailey–Lovie Word Reading Chart [28] and the SKread Charts [50]. Both tests use unrelated words which, in the case of the SKread, are also interrupted by single letters. With such tests, it is thought that linguistic aspects are widely excluded because reading performance depends on word and letter recognition alone. The English SKread charts [50] were compared to the MNREAD charts, and the German SKread charts [105] were compared to the German version of the RADNER Reading Charts; the reading speed and number of errors were compared to the MNREAD charts at a print size of 8M (decimal: 0.05 at 40 cm) and with the RADNER Charts at 5M (decimal: 0.08 at 40 cm). With the SKread test, the reading speed was significantly slower, and the number of errors was considerably higher for normally sighted subjects as well as for patients with a maculopathy, when compared to those obtained with the MNREAD and RADNER charts. This result indicates that paragraphs using unrelated words are more difficult to read, because performance relies on visual criteria alone, without the help of the linguistic aspects of the text. The repeatability of the maximum reading speed was found to be high for both versions of SKread (test–retest with an interval of 1 to 2 minutes), when the mean reading speed of five paragraphs above the CPS was calculated in normally sighted subjects. However, the repeatability was not given for other reading parameters, such as reading acuity. Not all SKread paragraphs from the whole set were included in these comparative studies.

Reliability and validity analyses of reading charts were introduced into ophthalmology in 2004 [32] with the investigation of the test–retest reliability (test interval: 3 to 4 weeks), interchart reliability, and variance component analysis for several reading parameters obtained with the RADNER Reading Charts (using a Latin square design). This study also included Bland–Altman analyses. For the reading acuity, reading acuity score, maximum reading speed, and logMAR/logRAD ratio, good repeatability was demonstrated. The Pearson correlations were high between the reading parameters and charts. The Bland–Altman plots showed a high degree of agreement between the two test sessions and among the three reading charts. In addition, a variance component analysis was performed [32, 59]. These analyses revealed that the individuals (patients) were predominantly responsible for the variability of the results. The testing procedure had only a minor influence on the whole variance, indicating that the test is highly reproducible.

Some months later, Virgili et al. [61] investigated a group of children with the Italian version of the MNREAD Charts, and demonstrated a good coefficient of repeatability (test interval: same day) for the reading acuity, reading speed, and CPS. In 2009, Subramanian et al. [60] reported the coefficient of repeatability in visually impaired patients for the English version of the MNREAD Charts (test interval: the same day). As in the study of the RADNER Reading Charts [32], a Bland–Altman analysis was again performed. In 2011, Patel et al. [65] reported a considerably weaker coefficient of repeatability in visually impaired AMD patients investigated with the MNREAD Charts than had been found by Subramanian et al. [60]. The difference in the coefficient of repeatability for reading acuity was 0.3 vs 0.1 logMAR; for different methods of calculating the maximal reading speed, the coefficients of repeatability were 0.22 to 0.25 vs 0.1 logwpm, and for the CPS, the coefficients of repeatability were 0.44 to 0.67 vs 0.3 logMAR. Patel et al. [65] concluded that in addition to patient-related aspects of variability, the fact that many different examiners investigated the patients during a clinical research trial may have been another factor (a variant component analysis to test this hypothesis has not yet been performed). It therefore cannot be fully excluded that the time period between the test and the retest examinations had a higher influence on the coefficient of repeatability than expected (test interval: the same day in the study of Subramanian et al., and 6 weeks in the study of Patel et al.).

Although single sentences or short paragraphs of unrelated words have become the preferred choice for test items used in modern calibrated reading charts, the question of whether long paragraphs or single sentences should be used is still a matter of interest. One of the reasons a single-sentence construction was used for the RADNER Reading Charts was that it allowed the introduction of a stop criterion, enabling an examiner to analyze the reading performance from fully read test items and not just from partially read long paragraphs, as can occur when reading the full paragraph would be too time-consuming in a busy patient care unit. Another reason was that single sentences are less susceptible to subjective influences such as motivation, interest, and mind-wandering or difficulty in concentrating [42, 44–46, 48, 106]. In addition, single sentences made it possible to control linguistic aspects such as grammar and syntax and to keep the geometric proportions of the test items constant throughout the whole reading chart [30, 31, 59, 62]. This consistency makes it easier to control the reliability and accuracy of a reading chart with respect to research and clinical purposes [30–32, 56, 57]. When long paragraphs are used for reading charts, they have to be reduced in length when the text length exceeds the space limits because of increasing print size, reducing their comparability to paragraphs in smaller print size. In addition, it is difficult to develop long paragraphs that are statistically comparable [48, 63], since significant differences can appear between long paragraphs even when the construction of the paragraphs is equal in terms of grammar, syntax, word length, number of syllables per word, and number of characters [48]. On the other hand, long paragraphs are advantageous when the reading fluency of a patient has to be optimized, as for low-vision care when reading aids are prescribed. Longer paragraphs can also be advantageous for fitting multifocal contact lenses in order to examine how the near addition works over a longer reading period.

Nevertheless, the answer to the question of whether single sentences or long paragraphs should be used for reading tests depends on the patient’s needs, because reading speed depends on many different factors, including the visual properties of the eye, the difficulty of the text, the length of the words used, interest, motivation, and mind-wandering [42, 44–46, 48, 106]. In addition, as noted previously, we have shown that reading speed changes with age [48], and this finding has recently been confirmed by Calabrese et al. [99]. Thus, the reading fluency and speed of a person varies within a range of reading speeds, confined by several subjective and objective circumstances that include linguistic aspects of the reading material, such as difficulty, word length, grammar, and syntax [46, 48, 50, 58, 100, 107, 108]. In other words, there is not just one reading speed for a person; the person’s reading speed is dependent on the characteristics of the test items used [46, 48, 50, 56, 57, 108].

In summary, it seems to be evident that calibrated, standardized reading charts such as the Bailey–Lovie Reading Word Reading Charts [28], the Colenbrander Cards, the MNREAD Charts [29], the Oculus Reading Probe II, the SKread Charts [50], and the RADNER Reading Charts [30–32] facilitate international and clinical communication in the field of reading performance and functional vision. Calibrated reading charts are available in many languages and have become a valuable tool for comparative analyses of reading performance.
